# Monkeypox: experts give virus variants new names

**Published:** 2022-09

1.

**12 August 2022** - A group of global experts convened by WHO has agreed on new names for monkeypox virus variants, as part of ongoing efforts to align the names of the monkeypox disease, virus and variants – or clades – with current best practices. The experts agreed to name the clades using Roman numerals.

The monkeypox virus was named upon first discovery in 1958, before current best practices in naming diseases and viruses were adopted. Similarly for the name of the disease it causes. Major variants were identified by the geographic regions where they were known to circulate.

Current best practice is that newly-identified viruses, related disease, and virus variants should be given names with the aim to avoid causing offense to any cultural, social, national, regional, professional, or ethnic groups, and minimize any negative impact on trade, travel, tourism or animal welfare.

Disease: Assigning new names to existing diseases is the responsibility of WHO under the International Classification of Diseases and the WHO Family of International Health Related Classifications (WHO-FIC). WHO is holding an open consultation for a new disease name for monkeypox. Anyone wishing to propose new names can do so here (see ICD-11, Add proposals).

Virus: The naming of virus species is the responsibility of the International Committee on the Taxonomy of Viruses (ICTV), which has a process underway for the name of the monkeypox virus.

Variants/clades: The naming of variants for existing pathogens is normally the result of debate amongst scientists. In order to expedite agreement in the context of the current outbreak, WHO convened an ad hoc meeting on 8 August to enable virologists and public health experts to reach consensus on new terminology.

Experts in pox virology, evolutionary biology and representatives of research institutes from across the globe reviewed the phylogeny and nomenclature of known and new monkeypox virus variants or clades. They discussed the characteristics and evolution of monkeypox virus variants, their apparent phylogenetic and clinical differences, and potential consequences for public health and future virological and evolutionary research.

The group reached consensus on new nomenclature for the virus clades that is in line with best practices. They agreed on how the virus clades should be recorded and classified on genome sequence repository sites.

Consensus was reached to now refer to the former Congo Basin (Central African) clade as Clade one (I) and the former West African clade as Clade two (II). Additionally, it was agreed that the Clade II consists of two subclades.

The proper naming structure will be represented by a Roman numeral for the clade and a lower-case alphanumeric character for the subclades. Thus, the new naming convention comprises Clade I, Clade IIa and Clade IIb, with the latter referring primarily to the group of variants largely circulating in the 2022 global outbreak. The naming of lineages will be as proposed by scientists as the outbreak evolves. Experts will be reconvened as needed.

The new names for the clades should go into effect immediately while work continues on the disease and virus names.

Available from: https://www.who.int/news/item/12-08-2022-monkeypox--experts-give-virus-variants-new-names

